# Economic evaluation of caregiver interventions for children with developmental disabilities: A scoping review

**DOI:** 10.1371/journal.pgph.0003928

**Published:** 2025-06-30

**Authors:** Angela Kairu, Edwin Dzoro, Vibian Angwenyi, Charles Newton, Charlotte Hanlon, Rosa A. Hoekstra, Amina Abubakar, Edwine Barasa

**Affiliations:** 1 Health Economics Research Unit (HERU), KEMRI Wellcome Trust Research Programme, Nairobi, Kenya; 2 Aga Khan University, Institute for Human Development, Nairobi, Kenya; 3 Neuroscience Unit, KEMRI-Wellcome Trust Research Programme, Center for Geographic Medicine Research Coast, Kilifi, Kenya; 4 Department of Psychiatry, University of Oxford, Oxford, United Kingdom; 5 Division of Psychiatry, Centre for Clinical Brain Sciences University of Edinburgh, Edinburgh, United Kingdom; 6 Centre for Innovative Drug Development and Therapeutic Trials for Africa, College of Health Sciences, Addis Ababa University, Addis Ababa, Ethiopia; 7 Department of Psychiatry, WHO Collaborating Centre in Mental Health Research and Capacity Building, School of Medicine, College of Health Sciences, Addis Ababa University, Addis Ababa, Ethiopia; 8 Department of Psychology, Institute of Psychiatry, Psychology and Neuroscience, King’s College London, London, United Kingdom; 9 Nuffield Department of Medicine, Centre for Tropical Medicine and Global Health, University of Oxford, Oxford, United Kingdom; NIE: National Institute of Epidemiology, INDIA

## Abstract

Globally, families with children with developmental disabilities (DDs) experience challenges, including social isolation, stigma, and poverty, especially in low-income settings in Africa. Most children with DDs in Africa remain unidentified and receive no formal support. Caregiver interventions focusing on education and training for the carers/parents have been shown to be adaptable and low intensity in implementation, although the economic evidence is limited. This review aimed to describe the evidence and methodological aspects of economic evaluations for caregiver interventions for DDs. The Arksey and O’Malley framework was applied. Seven electronic databases, grey literature and cited references were systematically searched to identify eligible studies. published from 1993 to 2023. We assessed the quality of the included studies using the Drummond checklist. Data were systematically extracted, tabulated, and qualitatively synthesised using inductive thematic analysis. From 7811 articles, twenty studies all in high-income countries were included, and focused on caregiver interventions for autism spectrum disorder (n = 7), attention deficit hyperactivity disorder (ADHD) (n = 6), disruptive behaviour and behaviour problems with ADHD (n = 5), intellectual disabilities (n = 1) and language delay (n = 1). Economic evaluation analyses included cost effectiveness (n = 11), costing (n = 3), cost utility (n = 2), cost consequence (n = 1) cost benefit (n = 1), and combined analyses (n = 2). Nine studies reported the interventions as cost effective and five studies reported the intervention to be cost saving. The main methodological challenges were related to costing, outcome measurement in children and the appropriate time horizon for modelling. Caregiver interventions demonstrate cost-effectiveness, with the available evidence supporting the adoption of the interventions as a promising avenue to strengthen access and reduce the associated healthcare costs. The identified key methodological challenges highlighted further research areas. Prioritizing more economic evaluation studies in this area would inform decision-making on efficient resource allocation, promote inclusivity and equitable access to services for children with DDs.

## Introduction

Developmental disabilities (DDs) are a heterogenous group of conditions characterized by impairments that affect a child’s physical, learning, or behavioural functioning [1]. Globally, 53 million children under the age of 5 years have DDs such as sensory impairment, intellectual disability, epilepsy, and autism spectrum disorder (ASD) [2]. With the lack of improvement of the global burden of developmental disabilities over the last three decades [2], the socioemotional consequences, academic challenges and economic cost to society will be sustained or may worsen over time [3]. More specific to DDs, neurodevelopmental disorders (NDDs) occur in the developmental period and induce deficits that produce impairments of functioning (personal, academic, occupational or social) [4]. They include intellectual disability (ID); ASD; Attention-Deficit/Hyperactivity Disorder (ADHD); communication disorders; neurodevelopmental motor disorders; and specific learning disorders [4]. The prevalence of NDDs has rarely been assessed as a whole, with existing literature highlighting multimorbidity as a norm [3]. The majority of children with DDs lack access to care especially in Africa, due to inadequate capacity of skilled human resource [5,6]. With a focus on NDDs, substantial evidence shows that parents can learn skills to effectively improve their child’s development and positive behaviour [7].

Caregiver-focused interventions have been developed to train and educate parents/carers of children with developmental disabilities to support them in their child’s development [8]. Caregiver interventions are often centred on increasing parental responsiveness to improve the child’s behaviour and communication outcomes [9–12]. Parent skills training models have shown to be low-intensity in implementation due to less practitioner time requirement, the narrower focus of parenting strategies, and are adaptable for clinic, home, groups or individuals [13,14]. Further, interventions in early childhood are shown to have the strongest impact on improving the child’s long-term outcomes and parent outcomes [10–12]. Therefore, the need to explore the childhood specific strategies is important to reduce the overall economic burden of the illness. As such, the use of economic evaluations on health interventions and strategies for developmental disabilities has steadily gained interest. The economic evaluation data provides policy makers with information on the best value for money for the interventions [15]. Considering resource limitations, it is important that health interventions are effective in reducing the burden of disease. Economic evaluations can provide this information, and it is important to assess the current scope of evaluations of child specific interventions to inform future policy decisions.

Economic evaluation is an approach that contributes evidence on the economic costs and outcomes of health interventions to inform efficient resource allocation within a budget constraint. [16]. Policy strategies to decrease the impact of developmental disabilities are optimally effective when they are informed by evidence that determines how to improve efficacy, efficiency and cost-effectiveness of interventions, and their translation into clinical practice [17]. With a goal of maximizing health and well-being, economic analyses are needed alongside effectiveness evidence for policy makers to identify the best options for child and mental health resources [18]. Costs and cost-effectiveness data for caregiver interventions for children with DDs is scarce. Lamsal et al (2017) highlight few economic evaluations that have been conducted to value interventions for children with NDDs resulting from challenges in applying economic evaluation methodologies for this heterogenous population [1]. For instance, Penner et al (2015) conducted a cost effectiveness analysis on a parent delivered pre-diagnosis intervention for ASD and noted the limited choice of outcome measures for ASD and availability of this data for the analysis [19]. Similarly, Byford et al (2015) carried out a cost effectiveness analysis on a parent-directed intervention for pre-school children with ASD and highlighted the absence of a suitable generic measure and societal value for a unit improvement in the ASD score as a limitation to conducting similar studies [20]. Further, Rodgers et al (2020) conducted a review on early intensive behaviour interventions for autistic children and underlined limited effectiveness evidence which constrained economic evaluation analysis for the interventions [21]. A review by Kularatna et al (2022) identified twelve model based economic evaluations for care for NDDs, however none modelled the impact on families and caregiver [22]. Evidently, the unavailability of quality data is a major limitation of economic evaluation analyses for these interventions.

For childhood intervention programmes, some of the methodological challenges are related to cost and outcome measurement and valuation, the requirements for sensitivity analyses, the decision rules adopted by decision makers and the interpretation of results considering contextual factors [23,24]. Given these factors, there is a notable gap in literature that provides an overview of economic evaluations for caregiver interventions for children with DDs. The aim of this scoping review is to map the body of literature and describe the nature of evidence available on the economic evaluations for caregiver interventions for NDDs. Additionally, the review appraised the quality of studies included and discussed their methodological challenges and ways to mitigate them. By outlining the available evidence, we aim to inform the paediatric economic evaluation methodological approaches utilized and associated challenges and build on existing literature on interventions for child and adolescent mental healthcare.

## Methods

We applied the scoping review approach proposed by Arksey and O’Malley [25], and adhered to the Preferred Reporting Items for Systematic Reviews and Meta-Analyses extension for Scoping Reviews (PRISMA-ScR) guidelines for reporting this review. The review protocol was uploaded to Open Science Framework (OSF) https://osf.io/ymwr2.

### Search strategy and selection criteria

An extensive literature search was conducted using electronic databases that included PubMed, PsycINFO, Web of Science, the International Network of Agencies for Health Technology Assessment (INAHTA), Paediatric Economic Database Evaluation (PEDE), CINAHL, Econ Lit through EBSCO from January 1993 to December 2023. The period was selected to capture a comprehensive picture within a relevant time frame with focus on a recent period to ensure findings reflect the most up-to-date evidence. Further, we searched databases of grey literature specifically google scholar, and references of the included studies. The final search was conducted on September 18^th^, 2024. The search terms used in all search strategies were categorized into 4 blocks including: i) caregiver (parent, family); ii) interventions (e.g., training, programme, groups); iii) neurodevelopmental disorders (e.g., autism, intellectual disability); and iv) economic evaluation (e.g., cost utility analysis and cost effectiveness analysis). The details of the search concepts can be obtained from S1 and S2 Tables. All citations were imported into an electronic database (Endnote version X8) [26], where duplicates were removed. Studies were then uploaded into Rayyan (https://www.rayyan.ai/) for title and abstract screening. The inclusion criteria comprised of empirical studies that reported on an economic evaluation with a focus on caregiver interventions for neurodevelopmental disorders, in all geographical locations as summarized in Table 1. Studies that focused on physical and sensory impairments in children and studies that did not report on economic evaluation models of caregiver interventions for NDDs were excluded. Further, studies published beyond the time frame, were reviews, and in languages other than English were also excluded. The reason for exclusion of each study were noted. Two reviewers (AK and ED) independently screened titles and abstracts to assess relevance based on inclusion and exclusion criteria (Table 1). A third reviewer (EB) resolved any variations established. Abstracts included were then assessed for full-text inclusion. Full text articles eligible for inclusion were then selected for data extraction.

**Table 1:** Inclusion and exclusion criteria summary.

	Inclusion criteria	Exclusion criteria
P (Population):	Children with NDDs: impairments in social communication domains including autism spectrum disorder (ASD), attention deficit hyperactivity disorder (ADHD), intellectual disabilities, language communication	NDDs: impairments: physical and sensory impairments
I (Intervention):	Caregiver interventions for children with NDDs	Interventions for caregivers of adults with disabilities
C (Comparison):	Alternative interventions (as specified in the studies)	Not applicable
O (Outcome):	Economic outcome measures for caregivers and/or children with NDDs	
Study design:	Economic evaluations, empirical studies	Reviews
Others:	All countries, English	Non-English

### Data extraction

The data was extracted using a tailored Microsoft Excel worksheet and summarized in a narrative format: author/year, country, setting, population, type of DD, study design, intervention, comparator, and the economic evaluation methods reported guided by the Consolidated Health Economic Evaluation Reporting Standards (CHEERS) checklist [27], and the Gates Reference Case for Economic Evaluations [28]. The detailed CHEERS checklist and Gates Reference Case are available in S2 Checklist. The extraction sheet was piloted for completeness using five sample studies. Data extraction was undertaken by AK and ED. Discrepancies in the study selection and data extraction were resolved through discussion between two reviewers (AK, ED) and a third reviewer (EB).

### Data synthesis

Economic findings were synthesised and presented as a narrative summary in conjunction with a tabular summary. Our synthesis findings were presented as the article selection summary, quality assessment of the articles, an overview of studies key characteristics, description of the economic evaluations in the articles and their results, and methodological challenges encountered in economic evaluations of caregiver interventions for children with DDs.

### Quality assessment

The quality of the included economic evaluations was rated using the Drummond 10-point checklist [29]. The checklist guides on the critique of economic evaluations by considering: 1) the research question; 2) the study/intervention description; 3) the study design; 4) the identification, 5) measurement, and 6) valuation of costs and consequences; 7) application of discounting; 8) incremental analysis; 9) clear presentation of results with uncertainty and sensitivity analyses; and 10) discussion of results in the context of policy relevance and existing literature. Based on this, the studies were rated using a scale developed by Doran [30] with a potential score of 1 for each of the checklist items. The aggregate scores reflected an economic appraisal of poor quality (scores 1–3), average quality (scores 4–7) and good quality (scores 8–10). Authors AK and ED conducted independent quality appraisal of the included studies.

## Results

### Article selection

**Fig 1:**
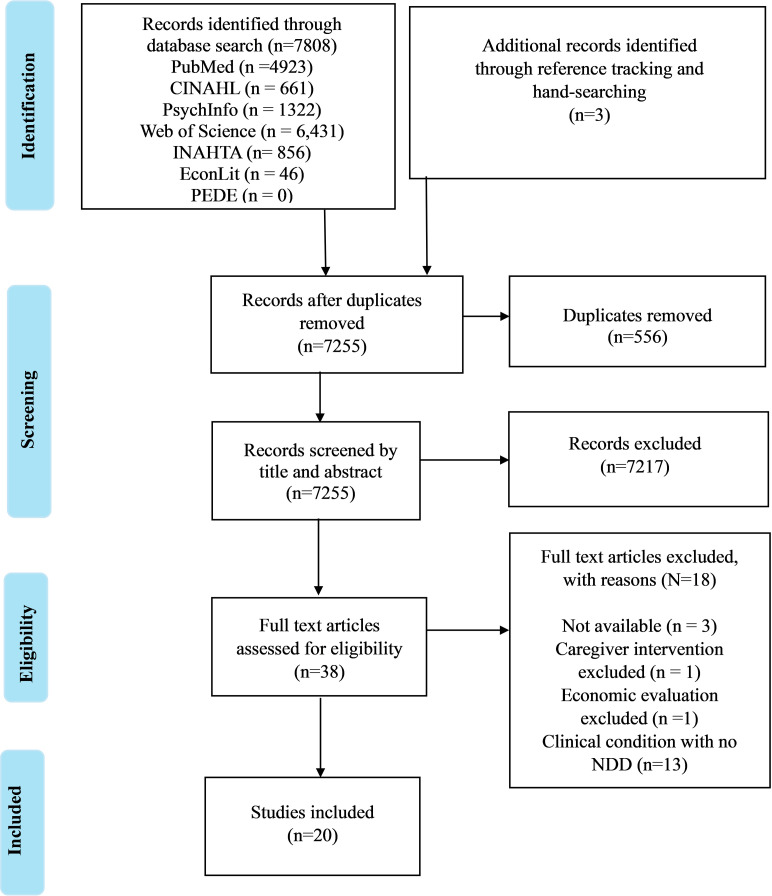
PRISMA flow chart of the study selection process.

The literature search identified 7,811 articles. After excluding duplicate studies, 7,255 studies remained for title and abstract screening. The screening based on title and abstract resulted in 38 articles eligible for full text screening. Most studies were excluded because they included a clinical condition with no NDD, they were not primary studies reporting economic evaluations results (e.g., reviews) or focused on clinical treatment rather than caregiver interventions for children with NDDs. After full-text screening, 20 studies were included for data extraction and quality assessment. Additional details are presented in the PRISMA flow diagram (Fig 1).

### Quality assessment

Most studies were classified as good quality (n = 16) [20, 31–42] and four studies were classified as average [43–46]. The most common reason for studies not receiving full points was due to lack of inclusion of discounting (n = 12) [20,31,32,36,37,39,42–47] and unclear presentation and discussion of results (n = 3) [34, 45, 46]. Details of quality scores for each study is presented in S3 Table.

### Overview of key characteristics

All the studies included were conducted in high income countries specifically UK (n = 7), Canada (n = 2), United States (n = 3), Australia (n = 1), Denmark (n = 1), Sweden (n = 3), Ireland (n = 1), Japan (n = 1), and multi-country (England, Ireland, Italy, Spain) (n = 1). Most of the studies focused on caregiver interventions for children with autism spectrum disorder (ASD) (n = 7), followed by attention deficit hyperactivity disorder (ADHD) (n = 6), disruptive behaviour with ADHD (n = 2), behavioural problems with ADHD (n = 3), intellectual disabilities (n = 1) and language delay (n = 1). The caregiver interventions were delivered through group format (n = 9), on individual basis (n = 8) or both platforms (n = 1). Two of the studies did not report on the intervention delivery method. Overall, the caregiver interventions targeted children ranging from 12 months to 12 years.

Of the reviewed studies, eleven reported a cost effectiveness analysis (CEA) [20,31,32,34,37,39,40,42,43,48,49], two studies carried out a cost utility analysis (CUA) [35, 46], one study performed a cost consequence analysis [50] and one study conducted a cost benefit analysis (CBA) [38]. However, two studies reported as CEAs [34, 40] were CUAs based on the measure of outcome as quality adjusted life years (QALYs) and one study conducted an additional CUA [48]. In addition to these, two studies conducted combined analyses as a cost analysis alongside a CEA [33], and a cost consequence analysis (CCA) alongside a CEA [41]. For partial economic evaluations, three studies conducted a costing analysis [36, 44, 45]. Fourteen were economic evaluations conducted alongside trials [20,31,32,34,36,40–46, 48, 49], whereas five studies were model based [33, 35, 38, 39, 50]. One study did not state the economic evaluation approach used [37].The time horizon for the majority of studies was relatively short, that is: 13 weeks for one study [43]; six to ten months for three studies [32, 36, 44]; and, one year for two studies [20, 48]. Eight studies extrapolated to a long-term period ranging from two years to 65 years [33, 35, 37–41, 50]. Six studies [31, 34, 42, 45, 46, 49] did not explicitly state the time horizon of the evaluations. In Table 2 we provide a detailed overview of the main characteristics of the included studies.

**Table 2:** Overview of the key characteristics of the included economic evaluation studies.

Serial number	Author & year	Developmental disability	Country	Study population (age group)	Study design (Model type)	Type of economic evaluation	Follow-up period (trials) or time horizon (models)
1	Royston et al [48], 2024	Intellectual disabilities	United Kingdom	2 years 6 months to 4 years 11 months	Randomised controlled trial	Cost effectiveness analysis (and cost utility analysis)	12 months
2	Shimabukuro et al [49], 2024	Attention-deficit hyperactivity disorder (ADHD)	Japan	6 to 12 years	Randomised controlled trial	Cost effectiveness analysis	Not reported
3	Segal et al [33], 2023	Autism Spectrum Disorder	Australia	1 year	Decision analytic model (Decision tree)	Cost analysis and Cost-effectiveness analysis	12 years
4	Kuklinski et al [37], 2023	Disruptive behaviour (ADHD and conduct disorder)	United States	Not reported	Economic evaluation	Cost effectiveness analysis	5 years
5	Tinelli et al [50], 2023	Autism Spectrum Disorder	Multi-country (England, Ireland, Italy, Spain)	2 years to 4 years 11 months	Economic evaluation	Cost consequence analysis	6 years
6	Tsiplova et al [36], 2022	Autism Spectrum Disorder	Canada	1 year 3 months to 3 years	Randomized comparative effectiveness trial	Cost analysis	24 weeks
7	Ferguson et al [45], 2022	Autism Spectrum Disorder	Ireland	2 to 6 years	Experimental research design (trial)	Cost analysis	Not reported
8	Charman et al [34], 2021	Autism Spectrum Disorder	United Kingdom	4 years to 8 years 11 months	Randomized controlled trial	Cost effectiveness analysis	Not reported
9	O’Farrelly et al [41], 2021	Behaviour problems	United Kingdom	1 to 3 years	Randomised controlled trial	Cost effectiveness analysis; cost consequence analysis	Short term analysis 24 months; Long term analysis beyond trial period
10	Scavenius et al [31], 2020	Attention-deficit hyperactivity disorder (ADHD)	Denmark	3 to 9 years	Randomized controlled trial	Cost effectiveness analysis	Not reported
11	Nystrand et al [40], 2020	Disruptive behaviour	Sweden	8 to 12 years	Randomized controlled trial	Cost effectiveness analysis	2 years
12	Nystrand et al [35], 2019	Behaviour problems (conduct disorder and ADHD)	Sweden	5 to 12 years	Decision analytic model (Markov)	Cost utility analysis	18 years
13	Nystrand et al [38], 2019	Behaviour problems (conduct disorder and ADHD)	Sweden	5 to 12 years	Decision analytic model (Markov)	Cost benefit analysis	2 years
14	Tran et al [43], 2018	Attention-deficit hyperactivity disorder (ADHD)	United States	7 to 11 years	Randomized controlled trial	Cost effectiveness analysis	13 weeks
15	Sonuga-Barke et al [46], 2017	Attention-deficit hyperactivity disorder (ADHD)	United Kingdom	2 years 9 months to 4 years 6 months	Multi-centre three-arm parallel randomised controlled	Cost analysis (initially planned to do a cost utility analysis)	Not reported
16	Sayal et al [32], 2016	Attention-deficit hyperactivity disorder (ADHD)	United Kingdom	4 to 8 years	Three arm randomized cluster trial	Cost effectiveness analysis	9 months (inferred)
17	Page et al [44], 2016	Attention-deficit hyperactivity disorder (ADHD)	United States	5 to 12 years	Randomized controlled trial	Cost analysis	10 months
18	Byford et al [20], 2015	Autism Spectrum Disorder	United Kingdom	2 years to 4 years 11 months	Multi centre randomised controlled trial	Cost effectiveness analysis	13 months
19	Penner et al [39], 2015	Autism Spectrum Disorder	Canada	1 year 3 months to 3 years	Decision analytic model (Decision tree)	Cost effectiveness analysis	65 years
20	Gibbard et al [42], 2004	Language delay	United Kingdom	1 year 10 months to 3 years	Randomised controlled trial	Cost effectiveness analysis	Not reported

### Economic evaluation methods

#### Cost estimates.

We summarized the cost estimates associated with caregiver interventions implementation and the associated healthcare costs for children with DDs. Nine studies conducted evaluations from one perspective only: four studies adopted a health provider’s perspective [31, 35, 37, 45]; two studies adopted a government (insurer) payer perspective [33, 41]; and three studies adopted a societal perspective [40, 42, 43]. Nine studies reported results from more than one perspective [20, 34, 36, 39, 42, 46, 48–50]. The remaining two studies did not explicitly state the perspective adopted [38, 44].

The health provider costs mainly reported on the intervention implementation costs, for instance training, personnel, overheads, treatment costs amongst others. Among these, only three studies [31, 37, 48] reported on both start-up and implementation costs of the caregiver interventions. Notably, Scavenius et al (2020) reported only financial costs and excluded opportunity costs such as income loss or other sector benefits, as they were not tangible costs of the intervention implementation [31]. Cost inclusion or exclusion may impact the study results by increasing the costs of the intervention, but at the same time may also reduce the incremental costs of the intervention. Four studies [35, 38, 46, 48] captured the costs of other sectors, i.e., education, social and crime services. Furthermore, Nystrand et al (2019; 2020) included cost offsets as the treatment costs avoided with the reduction of the disease prevalence incurred by the health sector as a cost input [35, 38]. For societal costs, four studies [39, 40, 48, 49] mainly included either direct costs (health service costs, medication, childcare, travel) or indirect costs (productivity loss), whereas eight studies [20, 32, 34, 36, 42, 43, 46, 50] comprehensively captured both direct and indirect costs. Most studies explicitly stated the currency and reported year used except six studies [20, 32, 34, 44-46]. Discounting was not necessary in the studies which collected costs over a one-year time horizon or less. However, where necessary due to a longer time horizon adopted by twelve of the included studies, a discount rate ranging from 2% to 6% per annum was applied with the exception of two studies [37, 39]. Therefore, the cost-effectiveness results reported may be inaccurate for both studies.

#### Health outcome measures.

We report key effectiveness outcomes of caregiver interventions and comparators in the eligible studies in Table 4. Except for the cost analysis, the most frequently reported health outcomes for cost utility analysis included four studies reporting on QALYs [32, 34, 40, 48], and one study studies on disability adjusted life years (DALYs) [35]. Of the studies that reported QALYs, the generic health related quality of life (HRQoL) measures used were Child Health Utility 9D (CHU-9D) and EuroQoL 5 dimensions Youth (EQ5D-Y). Health outcomes reported in nine studies applying cost effectiveness analysis were mostly per case reduction and per outcome score improvement amongst others [20, 31, 33, 37, 41-43, 48, 49]. O’Farrelly et al (2021) carried out a short term CEA and a long term CUA, but with the small differences in the QALYs and no difference in cost between the trial groups, only the short term CEA was reported with per outcome score improvement as the health outcome [41]. Further, Penner et al (2015) reported dependency-free life years (DFLYs) as the outcome measure [39], while Nystrand et al (2020) reported the net present value for the CBA [38]. Additional details included in each of the reviewed studies are listed in Table 3.

**Table 3:** Cost inputs associated with the caregiver interventions included in this review.

Serial number	Study	Intervention(s)	Study perspective	Cost components	Currency and year	Discount rate
1	Royston et al [48]	Stepping Stones Triple P (SSTP) programme	NHS, personal social services (PSS) and parents’/caregivers’	Set up costs: Trainee and trainer time (and preparation time), travel costs, attendance incentives and course materials Delivery costs: therapist time spent; any materials provided to parents Health and social care utilisation costs Expenditure on private use of treatments and therapies (out-of-pocket)	Sterling pounds 2018	Not reported
2	Shimabukuro et al [49]	Well Parent Japan (WPJ) programme	Societal and personal	Staff time (programme delivery and preparation), consumables and therapist supervision. Therapist travel costs were not included as it was expected that once implemented the intervention would be delivered within their regular place of work. Personal health care utilisation costs	Japanese Yen 2021	Not reported
3	Segal et al [33]	iBASIS–Video Interaction to Promote Positive Parenting (iBASIS-VIPP) intervention	Government (insurer)	Intervention costs: therapy, direct travel, therapy-related administration, and training and supervision. Treatment as usual: group sessions, therapy (occupational, physiotherapy, speech), paediatrician, psychologist. Expected downstream support costs funds to eligible participants to access a wide range of disability-related support services.	Australian Dollars, 2023	3%
4	Kuklinski et al [37]	First Step Next (FSN) intervention	Provider	Personnel costs, supplies (pre-intervention: teacher recruitment packets, student screening kits, student screening surveys. Intervention costs: manuals, timers, and supplemental books, food and snacks: Implementation support costs: the manual, a Jenga game, and an iPad.), overheads (20% of personnel costs)	United States Dollars, 2018	Not reported
5	Tinelli et al [50]	Preschool Autism Communication Trial (PACT) intervention	Service (public sector); societal	Service costs, (healthcare, education, social care) Living costs, care and assistance, education, healthcare, travel, training/support, and autism assistance dog. Societal costs: schooling and childcare costs, productivity losses (due to parents taking time off work to care for an autistic child), and informal (unpaid) care.	Euros 2020	3.5%
6	Tsiplova et al [36]	Parenting coaching intervention	Public payer and societal	Training facilitators time costs, coach labour time costs, medical specialist services, laboratory tests, out of pocket costs to parents, private provider costs reimbursed by insurance. Productivity loss.	Canadian Dollars, 2019	Not reported
7	Ferguson et al [45]	ABA- based parent training	Provider	Cost of purchasing and shipping the hardware, cost of BCBA direct “in session time”, cost of BCBA indirect data collection time Face-to-face project costs: cost of travel, indirect cost of travel time of BCBA, cost of BCBA direct “in session time”	Sterling Pounds (year not reported)	Not reported
8	Charman et al [34]	Predictive Parenting intervention	NHS^a^ and personal social service, public sector and societal	Intervention costs: administration and travel expenses. Service-related costs: salaries, overheads, medication Indirect costs: unpaid parent and carer support	Sterling Pounds (year not reported)	Not reported
9	O’Farrelly et al [41]	Video-feedback Intervention to promote Positive Parenting and Sensitive Discipline (VIPP-SD)	NHS^a^	Service-related costs: accommodation (e.g., foster care and supported housing), hospital services (e.g., inpatient stays, outpatient contacts, accident and emergency attendances), community-based health and social care services (e.g., contacts with GPs and clinical psychologists), and prescribed medication. Cost of delivering VIPP-SD: therapist salaries, training, supervision, equipment, on-costs (employers’ national insurance and superannuation contributions) and appropriate capital, administrative and managerial overheads.	Sterling Pounds, 2018	3.5%
10	Scavenius et al [31]	CiC^c^ is a manual-based program	Provider	Non-recurrent initial costs associated with CiC^c^ program development. Intervention setup costs: recruiting and educating volunteer trainers. Intervention running costs: venue rental, volunteers’ mileage, child minding services, supervision. Participants costs: Time-use (commuting time, training time) Opportunity costs (excluded)	Danish Kroner, 2015	Not reported
11	Nystrand et al [40]	Parent management Training (PMT) programme	Societal	Intervention costs: training cost, delivery cost (meetings/ sessions, material and venue costs, medication, special education services).	Swedish Krona, 2018	3%
12	Nystrand et al [35]	COPE Connect intervention. Comet intervention Incredible Years (IY) intervention	Provider	Training costs (training fees, practitioner allowance, hotel cost, trip cost, travel allowance); session running costs (practitioners cost, venue cost, materials, annual license fees) Cost offsets: health and education sectors	United States Dollars, 2015	3%
13	Nystrand et al [38]	Parent training programmes (COPE, Comet, Incredible Years, Connect)	Not reported	Intervention costs (training costs, travel cost for training, session running costs, annual license fees) Cost offsets, i.e., healthcare sector costs, education sector costs (in the form of assistants, smaller group sessions and special pedagogical support)	Euros, 2015	3%
14	Tran et al [43]	Child Life and Attention Skills (CLAS) program and parent-focused treatment (PFT)	Societal	Direct costs: personnel salaries and supplies Indirect costs: clinician travel time, parent and clinician time spent at training sessions, and parent time spent.	United States Dollars, 2011	Not reported
15	Sonuga-Barke et al [46]	New Forst Parenting Programme (NFPP)	Societal and NHS^a^	Service-related costs: care service use (health clinics, health visitors, GPs, paediatric and mental health services); extra educational provision (school nurses, educational psychologist); social services Non-service-related costs: parental time off work Direct non recurrent costs: course fees/ training Recurrent costs: material, preparation, supervision, therapist travel, administration, parent travel, creche, delivery Indirect costs: health services, family borne	Sterling Pounds (year not reported)	Not reported
16	Sayal et al [32]	Parent only intervention Combined parent-teacher intervention	Societal	Intervention costs: training, overheads and consumables, personnel costs Productivity loss costs	Sterling Pounds (year not reported)	Not reported
17	Page et al [44]	Group behavioral parent training	Not reported	Intervention direct costs: medication, personnel (physician, clinician, paraprofessional, teacher), fuel reimbursement. Indirect costs: parent’s time.	United States Dollars (year not reported)	Not reported
18	Byford et al [20]	Pre-school Autism Communication therapy	Societal and public sector	Direct costs: schooling and childcare costs, parental out of pocket expenditure (aids and adaptations to the home, training courses etc.). Indirect costs: productivity losses (time off work due to child’s autism) and informal (unpaid) care. Intervention costs: therapists salaries, overheads (capital, administrative and managerial), supervision, travel costs to home visits.	Sterling Pounds (year not reported)	Not reported
19	Penner et al [39]	Parent delivered Early Start Denver Model (ESDM)	Government payer and societal	Intervention costs (therapist, training cost), Provincial costs (special education services, disability program costs, vocational training, health service costs) Caregiver costs (excluding productivity loss)	Canadian Dollars, 2013	Not reported
20	Gibbard et al [42]	Parent based intervention (PBI)	Societal and provider	Costs included labour, administration, and overheads. Health personnel costs, hospital visit costs, capital and overheads, direct treatment costs. Out-of-pocket expenses borne by patients and their families (travel costs, clinic cost) and productivity loss costs.	Sterling Pounds, 1999	Not reported

^a^ NHS: National Health Service

^b^ GP: General Practitioner

^c^ CIC: Caring in Chaos

^d^ ABA:Applied Behaviour Analysis

^e^ IY: Incredible Years

^f^ PMT: Parent Management Training

**Table 4:** Clinical and economic outcomes of the economic evaluation studies included in this review.

Serial number	Study	Intervention(s)	Comparator	Clinical outcome	Economic outcome	Sensitivity analysis
**Cost-effectiveness analysis**						
1	Royston et al [48]	Stepping Stones Triple P (SSTP) programme	Treatment as usual	Child Behaviour Check List (CBCL) score	per CBCL score improvement	Univariate sensitivity analysis
2	Shimabukuro et al [49]	Well Parent Japan (WPJ) programme	Treatment as usual	Parent-domain parenting stress score	per reduction in parent-domain parenting stress score	Not reported
3	Segal et al [33]	iBASIS–Video Interaction to Promote Positive Parenting (iBASIS-VIPP) intervention	Treatment as usual	Incidence of ASD diagnosis	per lower incident case of diagnosed ASD	Univariate analyses and probabilistic analyses
4	Kuklinski et al [37]	First Step Next (FSN) intervention	Home based intervention (hB)	Clinical outcomes (ADHD^b^ vs CD^d^ vs ADHD^b^ plus CD^d^)	Per case reduction	Probabilistic sensitivity analysis
9	O’Farrelly et al [41]	Video-feedback Intervention to promote Positive Parenting and Sensitive Discipline (VIPP-SD)	Treatment as usual	Preschool Parental Account of Children’s Symptoms (PPACS) score	Per point improvement in PPACS score Quality adjusted life years (QALYs) (not reported)	Probabilistic sensitivity analysis and scenario analysis
10	Scavenius et al [31]	CiC^a^ is a manual-based program	Waitlist control group	Parenting competence measure by Parenting Sense Competence Scale (PSOC) Child functioning measured by Home Situations Questionnaire (HSQ)	per Cohen’s d effect sizes (ES) improvement (in the PSOC and HSQ)	Not reported
14	Tran et al [43]	Child Life and Attention Skills (CLAS) program and parent-focused treatment (PFT)	Treatment as usual	Not reported	Per resolved ADHD^b^-I case	One way and multiway sensitivity analysis
18	Byford et al [20]	Pre-school Autism Communication therapy	Treatment as usual	(ADOS-G) Autism Diagnostic Observation Schedule-Generic score	per ADOS-G score improvement	Not reported
20	Gibbard et al [42]	Parent based intervention (PBI)	Treatment as usual	Six measures on the child’s linguistic complexity from a single word level to that of using three-to-four-word utterances	Per change in measure score	Univariate sensitivity analysis
**Cost utility analysis**						
1	Royston et al [48]	Stepping Stones Triple P (SSTP) programme	Treatment as usual	Child Behaviour Check List (CBCL) score	Quality adjusted life years (QALYs)	Univariate analysis
8	Charman et al [34]	Predictive Parenting intervention	Psychoeducation	Behaviours That Challenge (BTC) per minute Autism Parenting Stress Index, Parent Efficacy scale	Quality adjusted life years (QALYs)	Univariate analysis
11	Nystrand et al [40]	Parent management Training (PMT) programme	Coping Power Programme and parent management Training (PMT) programme	Parent-rated Disruptive Behaviour Disorder rating scale (DBD-ODD)	Quality adjusted life years (QALYs)	Scenario analysis
12	Nystrand et al [35]	COPE Connect intervention. Comet intervention Incredible Years (IY) intervention	Waitlist control group	Swanson, Nolan and Pelham Scale (SNAP-IV) score Eyberg Child Behaviour Inventory (ECBI) score	Disability adjusted life years (DALYs)	Probabilistic sensitivity analysis and univariate sensitivity analyses
16	Sayal et al [32]	Parent only intervention Combined parent-teacher intervention	Waitlist control group	Parent- rated ADHD index Scores on the other parent-rated sub-scales and all the teacher-rated sub-scales of the Conners’ Rating Scale – Revised	Quality adjusted life years (QALYs)	Not reported
19	Penner et al [39]	Parent delivered Early Start Denver Model (ESDM)	Status Quo	Intelligence Quotient (IQ)	Dependency-free life years (DFLYs)	Multiple one-way sensitivity analysis
**Cost benefit analysis**						
13	Nystrand et al [38]	Parent training programmes (COPE, Comet, Incredible Years, Connect)	Waitlist control group	Swanson, Nolan and Pelham scale (SNAP-IV) scores	Net present value	Probabilistic sensitivity analysis
**Other economic evaluations**						
5	Tinelli et al [50]	Preschool Autism Communication Trial (PACT) intervention	Treatment as usual	Autism Diagnostic Observation Schedule-Generic (ADOS-G) score	N/A	Deterministic sensitivity analyses
6	Tsiplova et al [36]	Parenting coaching intervention	Enhanced community treatment	Not reported	N/A	Univariate analysis
7	Ferguson et al [45]	ABA^c^- based parent training	Face to face model training	Child affect measure	N/A	Not reported
15	Sonuga-Barke et al [46]	New Forst Parenting Programme (NFPP)	Incredible Years (IY)	Parent ratings of child’s ADHD symptoms Reduction of parent-rated ADHD symptoms	N/A	Not reported
17	Page et al [44]	Group behavioural parent training	Medication for ADHD^b^	Not reported	N/A	Univariate analyses

^a^ CIC: Caring in Chaos

^b^ ADHD: Attentive Deficit Hyperactivity Disorder

^c^ ABA:Applied Behaviour Analysis

^d^ CD; Conduct disorder

^e^ PMT: Parent Management Training

#### Sensitivity analysis.

At least one form of sensitivity analysis was carried out in 14 of the eligible studies as shown in Table 4. Scenario analysis was carried out in two studies [40, 41], univariate sensitivity analysis was carried out in eight studies [33–36, 42–44, 48], two studies [39, 43] carried out a multivariate sensitivity analysis, one study carried out a deterministic sensitivity analysis [50] and probabilistic sensitivity analysis was carried out in five studies [33, 35, 37, 38, 41]. Six studies did not conduct sensitivity analysis to explore uncertainties around their results [20, 31, 32, 45, 46, 49].

#### Statistical analysis.

Of the trial based economic evaluations, twelve of these studies further applied statistical analysis. Nine studies [20, 31, 32, 34, 36, 40, 42, 48, 49] applied regression models and bootstrapping methods to analyse the differences in costs and outcomes, whilst two studies [20, 48] adjusted for missing data. With trials being designed to assess the effectiveness of a new intervention for a specific population and CEAs/CUAs designed to account for benefits for even those not receiving a new health intervention, statistical analyses should be applied to address methodological challenges that may arise with the joint analysis.

#### Economic evaluation findings and policy suggestions.

Evidence of the findings of the economic evaluations of the caregiver interventions are summarised in Table 5. Of the 9 CEA studies and 5 CUA studies, four studies [20, 34, 40, 41] did not report outcomes using incremental cost effectiveness ratios (ICERs) due to small to no differences in the outcomes. Several of the studies reported the intervention as cost-effective (n = 9) and cost-saving (n = 5) especially when considering the long-term effects. A few of the studies found the intervention less likely to be cost effective. Charman et al (2021) found the predictive parenting intervention more costly and less cost-effective than its comparator [34]. Similarly, Byford et al (2015) found the combination of pre-school autism communication therapy (PACT) and usual treatment to have higher costs and no significant difference in outcomes [20]. Page et al (2016) found medication to be recommended for treatment of ADHD as it maximizes the societal cost compared to group behavioural therapy [44]. With the willingness to pay threshold being arbitrary, most of the studies used varied thresholds to determine cost-effectiveness of the interventions.

**Table 5:** Summary of findings and policy implications.

Serial number	Study	Intervention(s)	Type of economic evaluation	Costs	Incremental cost effectiveness ratio (ICER)	Willingness-to-pay (WTP) threshold	Policy implications
1	Royston et al [48]	Stepping Stones Triple P (SSTP) programme	Cost effectiveness analysis (and cost utility analysis)	Cost per participant: Intervention delivery £270 Training cost per participant £26	Cost saving of −£1057.88 per participant Very small differences in QALYs	£13,000, £20,000 and £30,000 per QALY gained	Stepping Stones Triple P is probably value for money to deliver, but decisions to roll this out as an alternative to existing parenting interventions or treatment as usual may be dependent on policymaker willingness to invest in early interventions to reduce behaviours that challenge.
2	Shimabukuro et al [49]	Well Parent Japan (WPJ) programme	Cost effectiveness analysis		6,707 (JPY) ($ 61.93) per QALY gained	10,000 JPY ($ 108.30) and 20,000 JPY ($ 216.60)	The cost-effectiveness analyses showed intervention costs to be modest and the programme cost-effective.
3	Segal et al [33]	iBASIS–Video Interaction to Promote Positive Parenting (iBASIS-VIPP) intervention	Cost analysis and Cost-effectiveness analysis	Total cost of iBASIS-VIPP delivery (including TAU services): A $5477 (US $3850) per child Total cost of TAU group: A $346 (US $243) per child Cost difference: A $5131 (US $3607) per child	Cost per reduction in an ASD diagnosis at age 3 years: A $37 181 (US $26 138) NPV cost savings: A $10 695 (US $7519) per child enrolled in iBASIS-VIPP (at 12 years age) Return of investment: A $3.08 (and US $3.08) for each dollar invested in iBASIS-VIPP.	Not stated	iBASIS-VIPP is likely highly cost-effective. iBASIS-VIPP likely represents a good-value societal investment. Identifying pre-emptive interventions that are efficacious and represent good value is an important input to resource allocation decisions.
4	Kuklinski et al [37]	First Step Next (FSN) intervention	Cost effectiveness analysis	Total cost of delivering FSN: US$3,226 per student Total cost of delivering combined intervention: US$3,801 per student	ADHD FSN: $12,433 per case reduction Combined intervention: $8,503 per case reduction CD FSN: $14,661 per case reduction Combined intervention: $11,051 per case reduction ADHD plus CD FSN: $16,909 per case reduction Combined intervention: $12,131 per case reduction	Varied	The combined intervention was more cost effective. Improvement in comorbid ADHD and CD was the costliest to achieve, followed by CD, and then ADHD.
5	Tinelli et al [50]	Preschool Autism Communication Trial (PACT) intervention	Cost consequence analysis	Total service perspective (health, education, and social services): England € 61,104; Ireland € 80,838; Italy € 31,204; Spain € 129,339 Total societal perspective (including parental productivity losses and informal care): England € 465,567; Ireland € 490,634; Italy € 366,299; Spain € 368,496.	N/A	N/A	PACT is cost-saving over time from a societal perspective, even though we confirmed that, at 13 months post-delivery, PACT is more expensive than usual treatment (across all countries) when given to preschool autistic children. After 6 years, we found that PACT has lower costs than usual treatment in terms of unpaid care provided by parents (in all countries).
6	Tsiplova et al [36]	Parenting coaching intervention	Cost analysis	Public payer PC group cost: $6594 per family ECT group cost: $4079 per family Mean incremental difference: $2515 Societal PC group cost: $23,925 ECT group cost: $16,931 Mean incremental difference: $6994	N/A	N/A	The PACE Coaching project demonstrated that a parent coaching intervention can be successfully implemented in a community setting. The present cost analysis identified and measured major cost components of the coaching intervention which could be valuable for public funding and planning decisions to serve this population
7	Ferguson et al [45]	ABA- based parent training	Cost analysis	Total project cost: £1,509.3 per parent	N/A	N/A	A telehealth training platform should be considered by behaviour analysts to expand their knowledge and increase the reach of their services, which could have benefits for parents who are not able to avail of training locally. Additional cost savings identified highlight the potential of a telehealth platform for service provision.
8	Charman et al [34]	Predictive Parenting intervention	Cost effectiveness analysis	Predictive Parenting Total NHS/PSS costs: £789.77 Total public sector costs: £1,393.42 Total societal costs: £3,092.87 Psychoeducation Total NHS/PSS costs: £434.48 Total public sector costs: £700.19 Total societal costs: £2,181.74	No difference in QALYs	£30,000 per QALY gain	Predictive Parenting was more expensive than Psychoeducation, with a low probability of being more cost-effective.
9	O’Farrelly et al [41]	Video-feedback Intervention to promote Positive Parenting and Sensitive Discipline (VIPP-SD)	Cost effectiveness analysis; cost consequence analysis	5 month follow up VIPP-SD intervention total costs: £1827.55 Usual care total costs: £510.18 24 month follow up VIPP-SD intervention total costs: £ 3131.93 Usual care total costs: £ 1525.38	Very small differences in QALYs	> £800 for a 1-point improvement	VIPP-SD is more costly and more effective than usual care. PPACS is not associated with a WTP threshold to support decision-making (compared with QALYs with a NICE WTP threshold of £20,000–30,000 per QALY), it is not possible to come to any firm conclusion about the relative cost-effectiveness of VIPP-SD in the short term.
10	Scavenius et al [31]	CiC^c^ is a manual-based program	Cost effectiveness analysis	Initial development costs $91,916 (671,265 DKK) Setup cost of training and running costs $67,648 (494,034 DKK) Cost per treated child $1,178 (8,601 DKK)	$1,707 (12,465 DKK) per effect size in PSOC $3,272 (23,892 DKK) per effect size in HSQ	$4,117 per family for one effect size in PSOC score	CiC may be a cost-effective alternative for improving parental competence, compared to other BPT programs.
11	Nystrand et al [40]	Parent management Training (PMT) programme	Cost effectiveness analysis	CPP intervention total cost per child: $584	Small difference in QALY gains	Varied	The results show that stacking treatments such as child-CBT and PMT may be money well spent compared to PMT only. Although costs are relatively small for the child component, investment in delivering both PMT and CPP depends on the willingness-to-pay for such a prioritisation.
12	Nystrand et al [35]	COPE Connect intervention. Comet intervention Incredible Years (IY) intervention	Cost utility analysis	Total cost per child Comet: $931 Connect: $334 Incredible Years: $1302 COPE: $478 Bibliotherapy: $14	Comet: $972 per DALY averted Connect: dominant Incredible Years: $1224 per DALY averted COPE: dominant Bibliotherapy: dominant	US$ 15,000 per DALY	Parenting interventions are cost-effective in the longer run in comparison to a waitlist control. Bibliotherapy or COPE are the most efficient options when comparing interventions to one another. Optimal decision for investment should be based on budget considerations and priority settings.
13	Nystrand et al [38]	Parent training programmes (COPE, Comet, Incredible Years, Connect)	Cost benefit analysis	Intervention costs per child Comet: € 817 Connect: € 295 Incredible Years: € 1142 COPE: € 417 Self-help book: € 13	Benefit cost ratio Comet: € 7 Connect: € 10.61 Incredible Years: € 5.96 COPE: € 15.80 Self-help book: € 328.04	Not stated	All the evaluated interventions within this study appear to be of good value-for money and yield substantial societal returns when adapted to various community populations, evidence that is relevant for local decision-making.
14	Tran et al [43]	Child Life and Attention Skills (CLAS) program and parent-focused treatment (PFT)	Cost effectiveness analysis	Total cost per patient CLAS: $1559 PFT: $710 TAU: $0 Incremental cost per patient PFT: $710 CLAS: $1559 (compared to TAU)	CLAS versus TAU $3997 per disordered case PFT versus TAU $3227 per disordered case CLAS versus PFT $4994 per disordered case	Not stated	Nonpharmacological behavioural interventions can be viable, cost-effective treatment options for children with ADHD-I. Future investigations are needed to evaluate cost-effectiveness of treatment for improving specific impairments associated with ADHD-I.
15	Sonuga-Barke et al [46]	New Forst Parenting Programme (NFPP)	Cost analysis (initially planned to do a cost utility analysis)	Total NFPP cost per family: £1591 Total IY cost per family: £2103 Cost difference: £512 NFPP intervention costs: £1081 IY intervention costs: £1569	N/A	N/A	The finding that NFPP may be less costly than IY supports a revision of NICE’s recommendations in favour of group rather than individual services. Both individually delivered and group-based PT should be made available to families of children with preschool ADHD.
16	Sayal et al [32]	Parent only intervention Combined parent-teacher intervention	Cost effectiveness analysis	Parent-only intervention £90 Combined interventions £107 Incremental cost parent-only programme: £73 Incremental cost of the combined programme: £123	Parent-only intervention: £29 per one point improvement in the ADHD index Combined intervention: £134 per one point improvement in the ADHD index	£31 per one-point improvement in the ADHD index	For children at risk of ADHD, an intervention programme for parents and teachers was not associated with improvement in core ADHD symptoms. The parent-only intervention programme suggested some potential for cost-effectiveness
17	Page et al [44]	Group behavioral parent training	Cost analysis	Behavior modification (group parent training) cost: $961 Low dose of stimulant medication cost: $1689	N/A	N/A	Medication is more likely be recommended than behavioural treatment and used as a first line treatment for ADHD, despite its greater cost, and this approach maximizes the societal cost of ADHD treatments.
18	Byford et al [20]	Pre-school Autism Communication therapy	Cost effectiveness analysis	Total cost PACT intervention: £4,105 per child PACT + TAU Total health, education and social service costs: £6,539 per child Total societal cost: £57919 TAU Total health, education and social service costs: £2050 per child Total societal cost: £56534	Not explicitly stated	Varied	PACT is associated with significantly greater costs and no significant difference in outcome, and thus should not be recommended as a cost-effective in addition to TAU. PACT plus TAU would only have a higher probability of being cost-effective compared to TAU alone if society is willing to pay extra for improvements in outcome.
19	Penner et al [39]	Parent delivered Early Start Denver Model (ESDM)	Cost effectiveness analysis	Provincial perspective costs Status Quo (SQ): $186,000 per person ESDM-Parent Delivered model (ESDM-PD): $178,000 per person ESDM Intensive model (ESDM-I): $199,000 per person	ESDM-I vs SQ: $23,000/DFLY ESDM-I vs ESDM-PD: $58,000/DFLY	Varied	Caregiver costs were a significant driver in cost-effectiveness estimates; consequently, from a societal perspective the pre-diagnosis intensive ESDM generated both cost-savings and enhanced outcomes relative to both the status quo and pre-diagnosis parent-delivered ESDM.
20	Gibbard et al [42]	Parent based intervention (PBI)	Cost effectiveness analysis	Cost per child PBI: £96 General care: £80.83 Cost per parent PBI: £31.80 General care: £5.78	£10.97 per score gained	Not explicitly stated	The results of this study are supportive of the effectiveness of this parent intervention programme, employing the setting of specific linguistic targets within the child’s naturalistic setting. This method of intervention also would not incur significant increases in cost per outcome gained over current practice and may, with modifications, result in cost savings.

## Discussion

This review identified economic evaluations of caregiver interventions for children with DDs and synthesized evidence on the methods adopted and their associated challenges. Twenty studies were identified that carried out both partial and full economic evaluations. Most of the studies evaluated interventions delivered through group programmes (47%) compared to individual based platforms (41%). One striking feature of this review is the heterogeneity of the economic evaluation approaches presented. The majority of studies (70%) were trial based economic evaluations, which are considered suitable for inferring cost-effectiveness for interventions with no prior evidence [51]. Although many of the included economic evaluations were rated average or good, many had methodological limitations, emphasising the challenges often faced in the conduct of economic evaluations of caregiver interventions for children with DDs. A few factors impact on the validity of conclusions drawn in economic evaluation such as accuracy of cost information, choice of discount rate, scope, modelling and time horizon and perspective. However, three main methodological challenges were identified from the literature as representative of the methodological issues more specific to conducting economic evaluations of caregiver interventions for young populations including time horizon analysis, measuring and valuing costs and measuring health outcomes.

Caregiver interventions for developmental disorders may result in long-term benefits and the choice of time horizon analysis should aim to capture all meaningful costs and outcomes. In our review seven studies extrapolated data over a one-year time horizon. One trial based economic evaluation study [43], modelled shorter timeframes due to lack of published data. However, the intervention showed a sustained effected with indication of it being even more cost-effective in later years of life. Similarly, O’Farrelly et al (2021) found that the short time horizon was a limitation as the participants were less likely to incur costs for service use relating to behavioural problems during the preschool period but instead when the child is older [41]. Current trial based economic evaluations have limited time horizons, often less than one year. Extending the time horizon of economic evaluations may lead to more favourable estimates [52], especially for interventions with long-term effects, as in the case of caregiver interventions. Two studies [39, 41] reported that the use of multiple data sources to extrapolate results over longer periods and to different population should be used with caution because this would require many assumptions and huge uncertainties which would likely produce poor estimates of the results. The uncertainties of parameters contribute to the overall model uncertainties whose potential impact can be assessed through scenario and sensitivity analyses, which are important considerations for economic evaluations [22]. Modelling studies may help address some of the issues of RCTs with longer period projections of the cost estimates and outcomes, and should use available evidence of real-world data and assumptions. Our review identified only four studies, which applied a decision analytical modelling approach such as decision trees or Markov models. Within any economic evaluation modelling, there is a balance between adequately simplifying complex health states and interventions to allow model parameterisation, while avoiding oversimplification to the extent that findings are not representative of the real-world context [22]. Even with diverseness of the methods applied, more than half (64%) of the reviewed studies found that the caregiver interventions were cost saving or cost-effective, while improving quality of life for the children and parents.

The studies reported varied levels of details of cost components used in the economic evaluations, with most studies including the major components as start-up and implementation costs. A few (18%) of the reviewed studies included costs from other sectors or cost offsets as important cross cutting and relevant costs which significantly contribute to wholistic care of children with DDs. Children with DDs require various services often extending beyond healthcare, for instance social services, education, rehabilitation, and criminal and justice systems [53]. Suhrcke et al (2008) conducted a systematic review demonstrating that for child and adolescent mental illness, only 6% of costs accounted for healthcare, with majority of the costs in other sectors (i.e., social services, education, productivity) [53]. Moreover, the service needs for children with DDs changes with the setting and age during their lifetime [24]. Therefore, quantifying service utilization for an economic evaluation of caregiver interventions may be challenging.

A prominent challenge related to cost measurement and valuation was the cost categories included. The studies identified exclusion of important cost inputs that would over- or underestimate the cost-effectiveness outcome. For societal costs, Scavenius et al (2020) did not account for productivity loss costs [31], and Nystrand et al (2019) did not include caregiver health and well-being costs and the costs of behaviour problem consequences in adulthood which would be significant cost offsets in the long term [35]. Regarding provider costs, Tsiplova et al (2022) excluded certain costs such as intervention development costs, and training costs (materials, coaches travel expenses, coach training) [36], and Nystrand et al (2020) included only medication costs in the healthcare resource use [38]. The accuracy of resources measurement was potentially limited by self-reporting of resource use for instance informal care and parent time costs in two studies [20, 43]. Another challenge noted was the lack of data which limited the costs included in the analysis. For instance, one study [43] was unable to assess all costs necessary for a full societal approach, another study [35] applied a narrow costing perspective, which likely missed different social impacts, because mental health interventions may have a broader societal impact, and interact with other sectors such as education, social services, justice, and voluntary sectors. Also, one other study [37] used a limited health system perspective and only cost offsets for one episode of care.

Notably, Penner et al (2015) found that caregiver costs were a significant cost driver in the cost-effectiveness estimates which further highlights the importance of accounting for all relevant costs [39].While inclusion of such costs may increase the intervention cost, the incremental costs of the intervention may also reduce. Adopting a narrower costing perspective may fail to incorporate other relevant sectors, which may bias study findings when most service utilization is beyond the healthcare system [1]. The economic benefits that accrued over time are realized not only by the participant and family involved, but also by other sectors and society in general [42]. In addition, the impact on productivity for caregivers may be considered in terms of time spent at work, and more often primary caregivers of children with DDs reduce working hours, give up work or change jobs to provide care [1]. Two main challenges with quantifying and valuing productivity are differentiating hours of care provided related to the DD compared to usual care and measuring productivity for children for their future earnings [54]. One of the reviewed studies [31] excluded productivity costs of parents and indicated possible underestimation, whereas two studies [35, 43] included parent time costs which added value to the findings. Although including productivity costs requires careful consideration, failure to capture productivity costs may potentially underestimate the resources use and effectiveness of the intervention [1].

Most of the included studies used cost effectiveness methods. Although informative, CEAs measure condition specific outcomes that are not directly comparable to other interventions targeting the same related problems or those across different diagnostic areas. In addition, the use of clinical measures undermines the likelihood of finding improvements that may be relevant to general well-being and everyday life, for instance improvement in quality of life. This is a key aspect of caregiver interventions that may have an impact on different areas of the children’s and parents’ lives. Therefore, cost utility studies which measure health outcomes with a generic health status that considers both mortality and the quality of life with morbidity would be preferred. In this review, only eight studies conducted CUAs with QALYs, DALYs and DFLYs used as the outcome measures. Measuring health related quality of life in children remains contentious because of the descriptive system or valuation perspective. For instance, measuring effectiveness of DDs is challenging and involves assessment of physical, behavioural, psychological, cognitive and social domains [1]. In this regard, our review highlighted methodological challenges towards measuring QALYs and DALYs in mental health and for the younger population.

First, the use of DALYs as an outcome may be limited by data availability on disease weights for specific DDs and the severity levels. For instance, Sampaio et al (2015) and Nystrand et al (2019) noted that conduct disorder (CD) doesn’t have differential severity weights, and significant assumptions on the disorder distribution and disability weights would be required to model changes in disorder severity [35, 55]. Consequently, children with some levels of CD who would benefit from the intervention and the benefits incurred by parents, siblings and teachers were excluded from the analysis, which would likely underestimate the health gains attributed to the reduction in disorder severity [55]. A better approach would specify weights based on disorder severity as a reflection of heterogeneity in health [56]. Second, the use of QALYs and the maximization approach to resource allocation in relation to mental health remains a contentious and understudied area [57]. Specifically, QALY measures may be “mis-valued”, insensitive and not specific to all mental health issues. Nonetheless, in failing to use QALYs there is risk of marginalization of mental health interventions in priority setting and budgeting processes. Third, there are challenges in methods of measuring and valuing health related quality of life in children, which have been studied to a limited extent than the adult population. Mihalopolous et al (2015) highlighted that the study was limited by the availability of adult population parameters which would not be representative of anxiety problems and the disease duration estimates in young children [58]. Based on the other health outcome measures applied in two studies [20, 41] it was noted that QALYs measures have mostly been applied in adult populations and may not be representative of the younger populations. In a recent review, some of the challenges highlighted included how to elicit informed preferences, the generation and use of combined adult and adolescent preferences and the appropriateness and acceptability of valuation tasks for children and adolescents [59]. Sayal et al (2016) utilized two measures CHU 9D and EQ5D-Y which showed slight variance in results likely from the differences in the valuation processes employed [32]. One study [35] used parental proxy rather than self-reported outcomes, which may be associated with misreporting. Thus, the use of different health related quality of life measures may show slight differences in the results, like one of the included studies [32]. Although the indirect approaches of utility measurement from multi-attribute utility instruments including EuroQol 5 dimensions (EQ-5D) and Health Utilities Index (HUI) are more appropriate to measure health status of children with DDs, deriving the parameter estimates can be challenging because of age-appropriateness, the domains, and methods to derive utilities [60]. Despite the challenges, there remains extensive opportunity for improvement of these estimates. Fourth, the application of utility values from other countries may be less accurate than country specific as highlighted in one of the reviewed studies [40]. In addition, there is limited data on utility values for some countries [38], or were condition-specific for instance per case reduction [39, 43], which limits the use of QALYs as outcome measures. The valuation of child and adolescent outcome measures remains a challenging research area that requires further empirical evidence to inform best practice.

### Strengths and limitations

A strength of this review is the application of the Arksey and O’Malley framework for scoping reviews which encourages collaborative engagement in the review process [25]. This involved identifying and developing the overarching research question, and the key terms to identify relevant studies. A notable limitation of this review was the relatively few studies to review with a strong focus on caregiver interventions for children with NDDs which may restrict generalizability of the findings while highlighting important areas for future research. While the search strategy was specific to NDDs, most papers focused on developmental disabilities all in high-income countries, evidently showing a literature gap. Costing and cost effectiveness data is highly context specific considering contextual factors that are important such as out of pocket costs, different health systems and different quality of life perspectives amongst others. This provides direction for future research across different contexts for example the African region.

## Conclusion

This review mapped economic evaluations of caregiver interventions for children with DDs and highlighted the gap in evidence and methodological challenges. Whilst economic evaluation analyses in this area are scarce, emerging data from common DDs was promising in the quest for cost saving and cost-effective interventions that improve quality of life of both the child and parent/caregiver. Caregiver interventions are a promising avenue to strengthen access and reduce costs associated with health services for children with DDs. This review has provided an overview of evidence on DDs which is a growing priority across many areas of paediatrics. Future research should consider the development of appropriate outcome measures and measurement of all relevant costs for this heterogenous population. Prioritizing more economic evaluation studies in this area would inform decision-making on efficient resource allocation, promote inclusivity and equitable access of services for children with DDs within the health system.

## Supporting information

S1 Table Search concepts and the corresponding key words used (concepts were combined using the Boolean operator “AND”). (DOCX)

S2 Table Search strategy- PubMed. (DOCX)

S3 Table Quality assessment of economic evaluations using the Drummond checklist. (DOCX)

S1 Checklist PRISMA-ScR checklist. (DOCX)

S2 Checklist CHEERS 2022 Checklist AND Gates Reference Case Principles. (DOCX)

S1 Data Scoping review data extraction table. (XLSX)
