# Supplementary material for: Economic evaluation of caregiver interventions for children with developmental disabilities: A scoping review
Source: PLOS Glob Public Health. 2025 Jun 30;5(6):e0003928. doi: 10.1371/journal.pgph.0003928 (PMC12208474; doi:10.1371/journal.pgph.0003928)
Supplement: S1 Table — (DOCX) [file pgph.0003928.s001.docx]

**S1 Table: Search concepts and the corresponding key words used (concepts were combined using the Boolean operator "AND")**

| Concept1 | Caregiver | caregiver mediated OR parent mediated OR family mediated OR parent based OR parents OR parent education OR parent psychoeducation |
| --- | --- | --- |
| Concept 2 | Training | intervention OR training OR programs OR programmes OR groups |
| Concept 3 | Developmental disabilities | developmental disorder or developmental disorders or disability or disabilities or disabled or handicap or handicapped or special education or special needs or inclusive education or developmental delay or developmental delays or neurodevelopmental disorder or neurodevelopmental disorders or autism or autistic or Asperger or childhood disintegrative or Rett or PDD or ASD or ADHD or attention deficit or hyperactive or hyperactivity or language disorder or language disorders or language impairment or intellectual disorder or intellectual disorders or intellectual deficit or intellectual impairment or mental retard or mental retardation or mental deficiency or mental subnormality or mentally retarded or Down syndrome or trisomy 21 or mongolism or fragile X or fetal alcohol or foetal alcohol or FAS or FASD or cerebral palsy or zika syndrome or Willliams syndrome or Tourette syndrome or Prader-Willi syndrome or anencephaly or phenylketonuria or microcephaly or epilepsy or cerebral palsy or paralysis or progressive disorders |
| Concept 4 | Economic evaluation terms | cost effectiveness analysis OR cost benefit analysis OR cost effective* OR cost utility OR cost benefit OR cost-benefit OR quality-adjusted life years OR disability adjusted life years OR cost* OR budget impact OR cost consequence OR cost-of-illness OR cost outcome description OR outcome description OR outcome analysis |
