# Supplementary material for: Economic evaluation of caregiver interventions for children with developmental disabilities: A scoping review
Source: PLOS Glob Public Health. 2025 Jun 30;5(6):e0003928. doi: 10.1371/journal.pgph.0003928 (PMC12208474; doi:10.1371/journal.pgph.0003928)
Supplement: S3 Table — (DOCX) [file pgph.0003928.s003.docx]

S3 Table: Quality assessment of economic evaluations using the Drummond checklist.

| **Study** | **Research question well defined?** | **Comprehensive description of alternatives?** | **Effectiveness of program established?** | **Important & relevant costs & consequences for each alternative identified?** | **Costs & consequences measured accurately & appropriately?** | **Costs & consequences valued credibly?** | **Costs & consequences adjusted for differential timing?** | **Incremental analysis of costs & consequences performed?** | **Allowance made for uncertainty in estimates?** | **Presentation & discussion of study results include all issues of concern to users?** | **Score** |
| --- | --- | --- | --- | --- | --- | --- | --- | --- | --- | --- | --- |
| Scavenius 2020 | **✓** | **✓** | **✓** | **✓** | **✓** | **✓** | **🗶** | **✓** | **🗶** | **✓** | **Good** |
| Sayal 2016 | **✓** | **✓** | **✓** | **✓** | **✓** | **✓** | **🗶** | **✓** | **🗶** | **✓** | **Good** |
| Segal 2023 | **✓** | **✓** | **✓** | **✓** | **✓** | **✓** | **✓** | **✓** | **✓** | **✓** | **Good** |
| Sonuga-Barke 2017 | **✓** | **✓** | **✓** | **✓** | **✓** | **✓** | **🗶** | **✓** | **🗶** | **🗶** | **Average** |
| Charman 2021 | **✓** | **✓** | **✓** | **✓** | **✓** | **✓** | **🗶** | **✓** | **✓** | **🗶** | **Good** |
| Byford 2015 | **✓** | **✓** | **✓** | **✓** | **✓** | **✓** | **🗶** | **✓** | **🗶** | **✓** | **Good** |
| Nystrand 2019 | **✓** | **✓** | **✓** | **✓** | **✓** | **✓** | **✓** | **✓** | **✓** | **✓** | **Good** |
| Tsiplova 2022 | **✓** | **✓** | **✓** | **✓** | **✓** | **✓** | **🗶** | **✓** | **✓** | **✓** | **Good** |
| Kuklinski 2023 | **✓** | **✓** | **✓** | **✓** | **✓** | **✓** | **🗶** | **✓** | **✓** | **✓** | **Good** |
| Nystrand 2019 | **✓** | **🗶** | **✓** | **✓** | **✓** | **✓** | **✓** | **✓** | **✓** | **✓** | **Good** |
| Tran 2018 | **✓** | **🗶** | **🗶** | **✓** | **✓** | **✓** | **🗶** | **✓** | **✓** | **✓** | **Average** |
| Page 2016 | **✓** | **🗶** | **🗶** | **✓** | **✓** | **✓** | **🗶** | **🗶** | **✓** | **✓** | **Average** |
| Penner 2015 | **✓** | **✓** | **✓** | **✓** | **✓** | **✓** | **🗶** | **✓** | **✓** | **✓** | **Good** |
| Nystrand 2020 | **✓** | **✓** | **✓** | **✓** | **✓** | **✓** | **✓** | **✓** | **✓** | **✓** | **Good** |
| Ferguson 2022 | **✓** | **✓** | **✓** | **✓** | **✓** | **🗶** | **🗶** | **🗶** | **🗶** | **🗶** | **Average** |
| O’Farrelly 2021 | **✓** | **✓** | **✓** | **✓** | **✓** | **✓** | **✓** | **✓** | **✓** | **✓** | **Good** |
| Gibbard 2004 | **✓** | **🗶** | **✓** | **✓** | **✓** | **✓** | **🗶** | **✓** | **✓** | **✓** | **Good** |
| Royston 2024 | **✓** | **✓** | **✓** | **✓** | **✓** | **✓** | **🗶** | **✓** | **✓** | **✓** | **Good** |
| Tinelli 2023 | **✓** | **✓** | **✓** | **🗶** | **🗶** | **✓** | **✓** | **✓** | **✓** | **✓** | **Good** |
| Shimabukuro 2024 | **✓** | **✓** | **✓** | **✓** | **✓** | **✓** | **🗶** | **✓** | **🗶** | **✓** | **Good** |
